# Roles for Ordered and Bulk Solvent in Ligand Recognition and Docking in Two Related Cavities

**DOI:** 10.1371/journal.pone.0069153

**Published:** 2013-07-18

**Authors:** Sarah Barelier, Sarah E. Boyce, Inbar Fish, Marcus Fischer, David B. Goodin, Brian K. Shoichet

**Affiliations:** 1 Department of Pharmaceutical Chemistry, University of California San Francisco, San Francisco, California, United States of America; 2 Department of Biochemistry and Molecular Biology, George S. Wise Faculty of Life Sciences, Tel-Aviv University, Ramat Aviv, Israel; 3 Leslie Dan Faculty of Pharmacy, University of Toronto, Toronto, Ontario, Canada; 4 Department of Chemistry, University of California Davis, Davis, California, United States of America; Universite de Sherbrooke, Canada

## Abstract

A key challenge in structure-based discovery is accounting for modulation of protein-ligand interactions by ordered and bulk solvent. To investigate this, we compared ligand binding to a buried cavity in Cytochrome *c* Peroxidase (CcP), where affinity is dominated by a single ionic interaction, versus a cavity variant partly opened to solvent by loop deletion. This opening had unexpected effects on ligand orientation, affinity, and ordered water structure. Some ligands lost over ten-fold in affinity and reoriented in the cavity, while others retained their geometries, formed new interactions with water networks, and improved affinity. To test our ability to discover new ligands against this opened site prospectively, a 534,000 fragment library was docked against the open cavity using two models of ligand solvation. Using an older solvation model that prioritized many neutral molecules, three such uncharged docking hits were tested, none of which was observed to bind; these molecules were not highly ranked by the new, context-dependent solvation score. Using this new method, another 15 highly-ranked molecules were tested for binding. In contrast to the previous result, 14 of these bound detectably, with affinities ranging from 8 µM to 2 mM. In crystal structures, four of these new ligands superposed well with the docking predictions but two did not, reflecting unanticipated interactions with newly ordered waters molecules. Comparing recognition between this open cavity and its buried analog begins to isolate the roles of ordered solvent in a system that lends itself readily to prospective testing and that may be broadly useful to the community.

## Introduction

Molecular docking is widely used to screen large libraries of molecules for those that will complement a site on a biological target. Whereas the technique has had important successes over the last decade [1]–[10], it retains several liabilities: it cannot predict binding affinities, nor even rank-order the affinities of diverse molecules. Consequently, docking is benchmarked for its ability to enrich ligands over non-binding decoy molecules [11] or, more compellingly, by prospective hit-rates (actives/tested). The retreat to these criteria reflects the entangled challenges that docking faces: it screens million-molecule libraries, and the molecules are diverse in chemotypes, topology, and physical properties. The diversity of these libraries negates one of the great equalizers of medicinal chemists: comparing differences in related series. Meanwhile, docking scoring functions must model ligand interactions in physically complicated binding sites with multiple residue types and strong, counter-balancing terms like electrostatic interactions, desolvation and hydrophobic burial, all in a condensed phase [12].

When confronted with complicated problems with entangled terms, investigators have often turned to simple model systems where these terms can be isolated: in genetics, this strategy has driven research in model organisms since Morgan in the 1920s [13]–[15], while in biophysics it has driven the development of small model proteins for understanding protein folding and stability, including Staphylococcal nuclease [16], barnase and barstar [17], and T4 lysozyme [18]. We and others have used small cavity sites as model systems to isolate particular energy terms in docking, analyzing one term at a time with different cavities. These cavities share several properties: they are all small (150 to 200 Å^3^), buried from bulk solvent, with hundreds to thousands of likely-but-untested ligands among our current libraries, binding may be readily tested by direct binding assays and crystallography, and each cavity site is dominated by one or two interaction terms. Thus, the L99A cavity mutant in T4 lysozyme is dominated by non-polar recognition, while the L99A/M102Q variant introduces a single carbonyl oxygen into this otherwise apolar site, and L99A/M102H further increases this cavity’s polarity [18]–[21]. Another type of cavity, the W191G mutant of Cytochrome *c* Peroxidase (CcP) is dominated by ion-pair interactions with Asp235 [22], [23]. Because of their simplicity, docking against these model cavities has revealed particular errors in our scoring functions and our representation of molecular properties, most often by the misprediction of molecules, which in these simple sites are often illuminating. Examples include the importance of using higher-level partial atomic charges for ligands [20], the challenges posed by decoy molecules when van der Waals repulsion terms are softened [24], the need to account for strain energy when modeling receptor flexibility [25], the trade-offs between optimizing geometric fidelity and ligand discovery [26], the consequences of neglecting ordered and especially bridging waters in the docking calculations [27], the challenges of correctly balancing van der Waals and electrostatic interaction terms in docking [21], and the opportunities and challenges for even the highest level of theory to predict binding affinities in these simple sites [28].

For all their advantages, the cavity sites leave important questions unaddressed, especially relating to the interaction with a bulk solvent interface, the higher dielectric boundary that it implies and, in many of the cavities, displacement of ordered waters – these are terms and challenges often encountered in biological targets. The failure to represent these terms owes to the buried nature of these cavities, which is typically a simplifying advantage of them, but does preclude a direct bulk water interface (though not an electrostatic interaction with it, of course [29]). We therefore looked for a cavity site that had an interface with bulk solvent but otherwise kept its qualities of simplicity, size, and dominance by a single interaction term.

**Figure 1:**
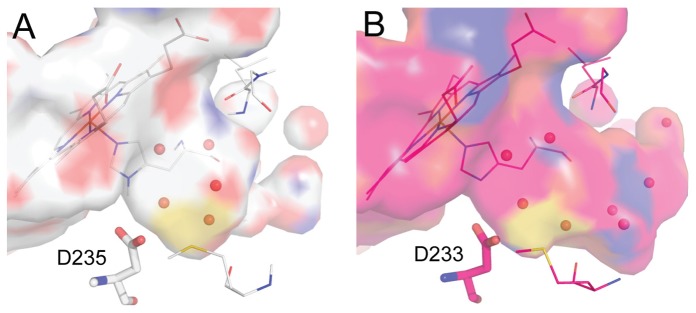
Comparison of the closed and open cavities. (A) The W191G cavity displays a closed and buried cavity that accommodates five ordered water molecules (PDB 1CMQ). (B) The Gateless cavity shows an open and larger pocket. Eight ordered water molecules extend from the back of the Gateless cavity, near Asp233, and out to solvent (PDB 1KXN).

We turned to a mutant of the CcP W191G cavity where the substitution Pro190→Gly has been made and residues Gly192 and Ala193 have been deleted (P190G/W191G/Δ192-3) [30]. These residues do not themselves directly interact with ligands but form a capping loop that seals off the original W191G cavity; their deletion opens this cavity to solvent. In crystal structures of the apo- and of the 2 ligand complexes determined before this study, this opening sequesters a chimney of eight ordered water molecules from the center of the active site to the bulk (**Figure 1**). In this new “Gateless” cavity we wished to investigate the following questions. First, how would ligands of the closed W191G cavity be affected by the opening to bulk solvent? In the closed cavity, small aryl cations like N-methyl-pyridine and thiophene-amidinium, which ion-pair with Asp235 (Asp233 in the Gateless mutant), had bound two to three log-orders better than neutral molecules like phenol and catechol. In the Gateless mutant one could imagine that the proximity to the bulk would diminish the affinity for mono-cations by increasing the effective dielectric or the solvation of the anionic Asp233, thus increasing competition between ligands and water. Empirically, such a loss in affinity has in fact been observed among three cationic ligands known for this cavity [30]. Counter-balancing this, the penalty for ligand desolvation might also be reduced, actually strengthening some affinities. Second, we wondered if a docking screen would track these changes – whatever they were – in the identities of the ligands it would predict, and how different models of ligand solvation, implemented in the docking method, would perform. Because this Gateless cavity remains relatively small, at ∼450 Å^3^, we anticipated many likely ligands in the ZINC library [31]. We therefore addressed these questions in a prospective docking screen, where the predictions were tested experimentally by binding affinity measurements and by X-ray crystallography.

## Results

### Comparison of Ligand Binding to the Closed and Open Cavities

**Figure 2:**
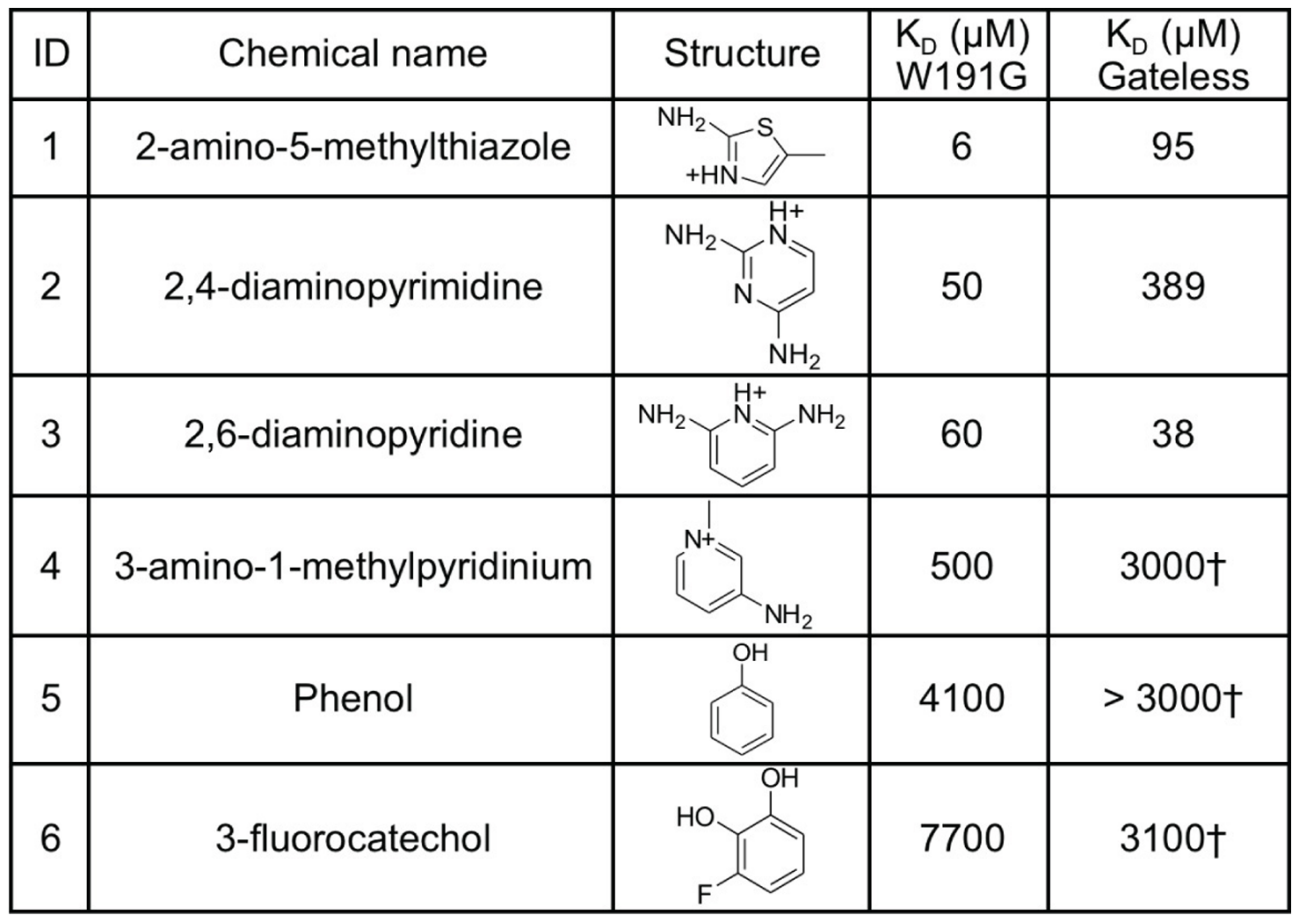
Comparison of binding affinities to the CcP W191G and Gateless cavities. ^†^Approximate KD determined by endpoint UV-Vis assay or partial ITC curves - assessment of these compounds was limited by solubility. ITC data for compounds **1**, **2**, **3**, **4** and **6** are available as supporting information (**Table S1**).

**Figure 3:**
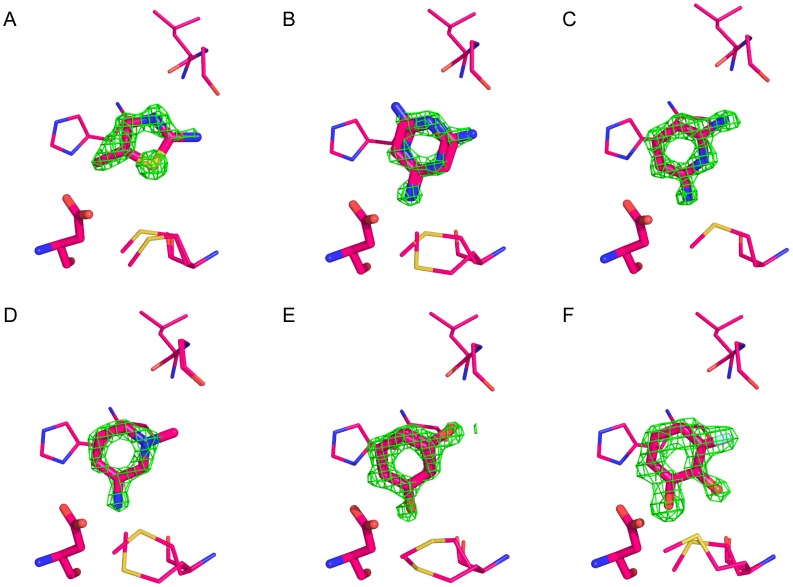
Electron density difference maps. Initial F_o_−F_c_ maps contoured at 3σ for (A) **1**, (B) **2**, (C) **3**, (D) **4**, (E) **5** and (F) **6**. PDB codes are as follows: **1** 1AEN/4JM5; **2** 2EUN/4JM6; **3** 2ANZ/4JM8; **4** 2EUO/4JM9; **5** 2AS3/4JMW; **6** 2AS4/4JMA.

**Figure 4:**
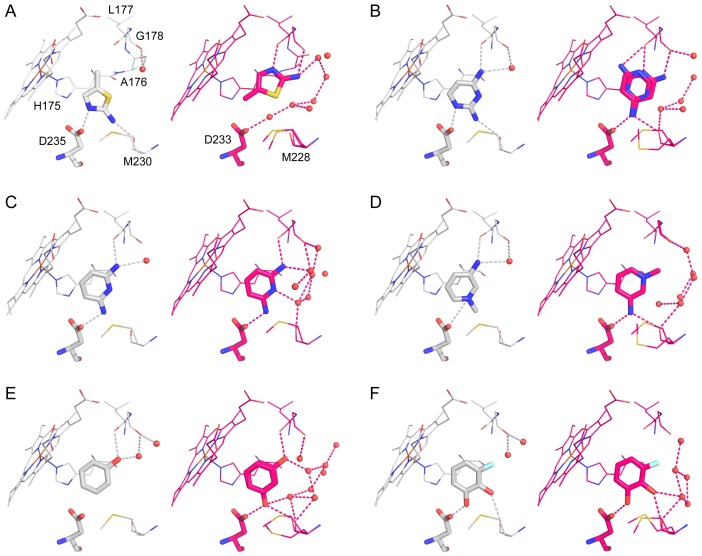
Crystallographic poses for six ligands in W191G (gray) and Gateless (pink). (A) 2-amino-5-methylthiazole (**1**). (B) 2,4-diaminopyrimidine (**2**). (C) 2,6-diaminopyridine (**3**). (D) 3-amino-1-methylpyridinium (**4**). (E) Phenol (**5**). (F) 3-fluorocatechol (**6**). PDB codes for the W191G/GA structures are as follows: **1** 1AEN/4JM5; **2** 2EUN/4JM6; **3** 2ANZ/4JM8; **4** 2EUO/4JM9; **5** 2AS3/4JMW; **6** 2AS4/4JMA.

Our first interest was to investigate the effect of opening the cavity to bulk solvent. Six known ligands of the buried W191G cavity were tested for binding to the Gateless cavity (P190G/W191G/Δ192-3) [30] by UV-Vis titration or by Isothermal Titration Calorimetry (ITC) (**Figure 2**, **Table S1** and **Figure S1**). To investigate these effects at atomic resolution, the six ligands were then crystallized in complex with the new Gateless cavity, with resolutions ranging from 1.19 to 1.60 Å (**Figure 3** and **Table S2**), and compared to their complexes with the W191G cavity (**Figure 4**).

In going from the W191G to Gateless cavities, three cationic compounds suffered a substantial loss in affinity: 2-amino-5-methylthiazole (**1**), 2,4-diaminopyrimidine (**2**) and 3-amino-1-methylpyridinium (**4**) (16-fold, 8-fold and 6-fold, respectively) (**Figure 2**). Conversely, the affinity of a fourth aryl cation, 2,6-diaminopyridine (**3**), was about 1.5-fold better in Gateless than in the closed W191G. The affinity of neutral phenol (**5**) was almost unchanged between the two cavities, while that of 3-fluorocatechol (**6**) improved 2-fold (from 7.7 mM in W191G to 3.1 mM in Gateless).

In the two cases where the affinity of the ligand increased upon opening the cavity to solvent (compounds **3** and **6**), we observed no change in ligand binding mode between the W191G and Gateless cavities (**Figure 4** **C** and **F**). In both Gateless complexes, the ligands participated, without reorientation, in an extensive water network created by the opening of the cavity to solvent. This new water network occupies the vacant space between the small ligands and the interface with bulk solvent and connects Gly178 to Met228. Phenol (**5)** also adopted the same geometry in W191G and Gateless, although a second binding mode was observed in the Gateless complex that had not been observed in W191G (**Figure 4** **E**). The new water network observed for compounds **3** and **6** was also observed for phenol. Conversely, compounds **1**, **2** and **4** underwent gross changes in their orientations in the Gateless versus the W191G cavity, and suffered substantial losses in affinity (from 1 to 1.6 kcal/mol). In the W191G/**1** complex, the ligand interacted with Asp235 and the backbone carbonyl of Met230 (**Figure 4** **A**). The same pattern of interactions was observed for the W191G/**2** complex, with an extra contact with Leu177 and a conserved water molecule (**Figure 4** **B**). In the Gateless/**1** complex, however, the ligand flipped by almost 180 degrees away from the Asp235 (now Asp233, owing to the residue deletion), opening this residue to solvation and to a direct interaction with the new water network (**Figure 4** **A**). Similarly, compound **2** bound to Gateless with 2 orientations, neither of which resembled the W191G binding mode (**Figure 4** **B**). In both, the nitrogen that is formally charged on the pyrimidine ring pointed away from Asp235(233), interacting with the Leu177 backbone instead. Here again, the new orientation appears to maximize interactions with the new water network at the cost of interacting with the anchoring aspartate, which is now more accessible to solvent. Finally, compound **4**, which ion-paired with Asp235 via its pyridinium nitrogen in W191G (**Figure 4** **D**), flipped to appose this same group with His175 in Gateless. This flip leads to more extensive interactions with the new water network for both the ligand and the aspartate, which though it did not directly interact with the new waters, was closer to them (**Figure 4** **D**).

In summary, for ligands that both maintain previous interactions with the aspartate and that make new interactions with the water network, affinity increases. Conversely, ligands that change their orientation when binding to the open cavity have weaker affinities. Naturally, these ligands do not bind weaker because they change orientations; rather, they change orientations because the geometry they had adopted in the closed cavity – where they typically form a direct salt-bridge to Asp235– has become higher in energy than an orientation that maximizes their interactions with the new water network. This is a point to which we return.

### Prospective Docking against the Open Cavity

To investigate the ability of docking to predict new ligands of the Gateless cavity, 534,000 molecules with molecular weight between 30 and 250 Da were docked against its structure. Using a variation of an receiver operator curve (ROC) that plots log_10_ of the percent of decoys found on the x-axis, which acts to up-weight early ligand enrichment, and that corrects for the enrichment seen at random, logAUC^32^, high enrichment of known ligands was observed (logAUC 42.56), with the three sub-millimolar ligands (**1**, **2** and **3**) in the top 1.4% of the database (**Figure S2**). Whereas the average molecular weight of the six known ligands for Gateless (**Figure 2**) is 112 Da, larger compounds dominated the top of the docking list, with an average molecular weight of 210 Da for the first 500 compounds (80% between 190 and 250 Da). Intriguingly, a substantial number of highly-ranked compounds were uncharged (>20% in the top 5000 molecules) **(Figure S3)**.

**Figure 5:**
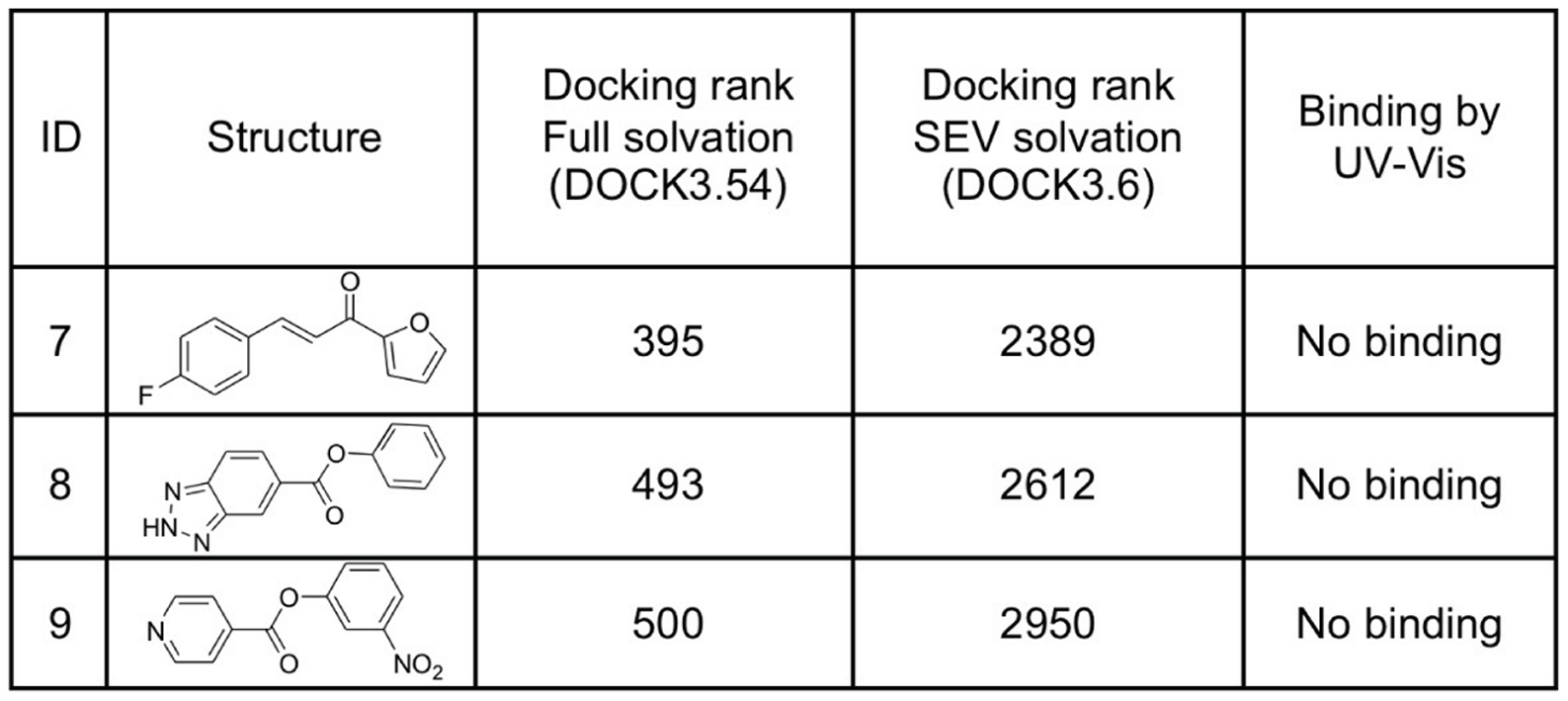
Three neutral compounds highly-ranked by the DOCK3.54 ligand solvation method [20], [74] do not bind detectably to the Gateless cavity. These ligands were de-prioritized by the Solvent-Excluded Volume ligand solvation method used in DOCK3.6. [32].

We were skeptical of this result, given the very modest affinities of phenol (**5**) and 3-fluorocatechol (**6**) for this site, and so turned to a Solvent-Excluded Volume (SEV) method of accounting for ligand desolvation in docking [32]. This method calculates the amount of solvent dielectric excluded by the volume of low-dielectric protein for any given configuration of a ligand in a binding site, using this value to calculate the ligand desolvation through a version of the Born equation. In this treatment a ligand is more or less desolvated depending on the overall volume of protein that surrounds it, and even a fully buried ligand retains an interaction with the bulk, remaining partially solvated, as is physically correct. This SEV method provides a better model than either considering a ligand fully desolvated on docking to a site–which over-penalizes it–or not desolvating it at all, which under-penalizes it, and does so in a physics-based manner consistent with the rest of the DOCK3.6 scoring function [32]. In retrospective studies, this SEV method had improved enrichments for sites where the solvent interface plays an important role [32] and also appeared to do so in a prospective screen [33]; this study represents the first test of the method, in a model system that enables detailed analysis of the results. Redocking the ZINC library with the SEV method, the fraction of neutral compounds in the top 5000 molecules of the docking list dropped to 3% (**Figure S3**) and enrichment for active compounds improved slightly (logAUC 44.34). This fit with our expectation that this site, though opened to solvent, would still be dominated by cationic ligand recognition. To actually test this, three neutral molecules, compounds **7**, **8** and **9**, that were prioritized by the older full desolvation method (ranked 395, 493 and 500) and de-prioritized by the SEV method (ranked 2389, 2612 and 2950) were tested for binding; none showed measurable affinity for the Gateless cavity at up to 1 mM concentration (**Figure 5**).

**Figure 6:**
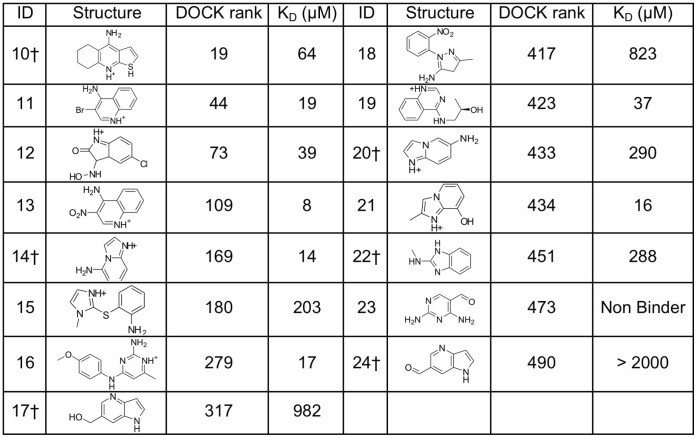
Binding affinities and DOCK ranks (Solvent-Excluded Volume solvation method) for compounds selected from a screen of 534,000 fragments against CcP Gateless cavity. ^†^ Crystal structures determined in complex with the CcP Gateless cavity.

**Figure 7:**
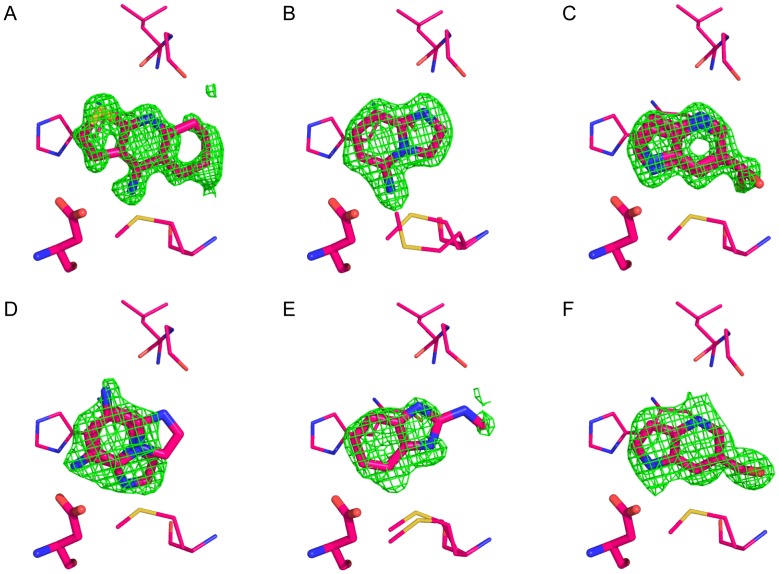
Electron density difference maps. Initial F_o_−F_c_ maps contoured at 3σ for (A) **10**, (B) **14**, (C) **17**, (D) **20**, (E) **22** and (F) **24**. PDB codes are as follows: **10** 4JMB; **14** 4JMS; **17** 4JMT; **20** JMV; **22** 4JMZ; **24** 4JN0.

**Figure 8:**
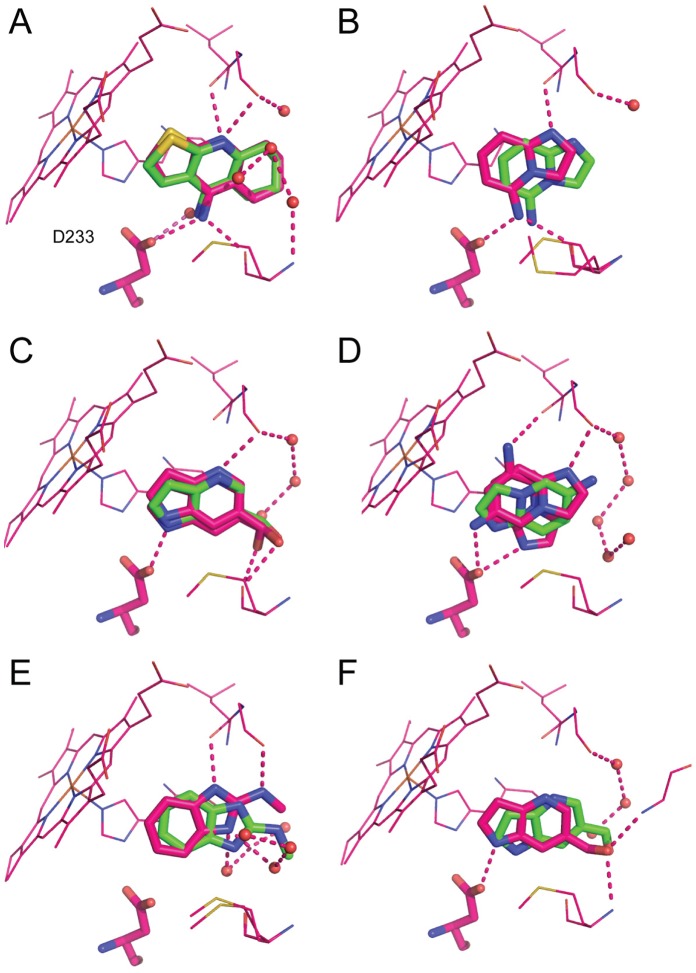
Superposition of the docking pose (green) and the crystallographic pose (pink) for six ligands prioritized by docking against the open Gateless cavity. Compounds (A) **10**. (B) **14**. (C) **17**. (D) **20**. (E) **22**. (F) **24**. All waters shown are from the co-complexed crystal structures. PDB codes are as follows: **10** 4JMB; **14** 4JMS; **17** 4JMT; **20** JMV; **22** 4JMZ; **24** 4JN0.

The Gateless cavity offered the first chance to test the new SEV solvation method prospectively. A further fifteen molecules, in addition to the three neutral molecules from the previous hit list, were selected from among the top 500 compounds of the SEV solvation-based hit list, or top 0.1% of the database screened, and tested for affinity. In addition to the docking score that ranked them among the top 500 molecules, these compounds were selected for favorable interactions with key binding site residues, such as Asp233, for chemotype diversity, and for compounds that were unburdened by known problems of the DOCK3.6 protocol and scoring, pricipally incorrect ionization and tautomerization states of the docked molecules, and occasionally high-internal energy conformations, as previously described^33^. Binding was detected for 14 of these 15 with affinities ranging from 8 to 982 µM (ligand efficiencies from 0.36 to 0.66) (**Figure 6**). Crystal structures of Gateless in complex with six of the new docking-predicted ligands were determined with resolutions ranging from 1.30 to 1.86 Å (**Figure 7** and **Table S2**). The structures of two ligand complexes, those of compounds **10** and **17,** superposed to within 0.5 Å of the docking prediction, three structures (**14**, **22** and **24**) did to within 1.4 Å of the docking prediction, and for one ligand (**20**) the docking pose was over 3 Å away from the crystallographic result (**Figure 8**). The crystallographic orientations of compounds **14** and **24** differed mainly by a translational shift, leading to a 1.1 Å r.m.s.d. between docking and crystallographic poses in both cases (**Figure 8** **B** and **F**). Intriguingly, the aldehyde in the docking pose of **24** was rotated by ∼90 degrees, but this variation did not affect the pose prediction. For compound **22**, the correspondence between docking and crystallographic pose was slightly worse (1.4 Å) (**Figure 8** **E**). In the docking pose, the ligand contacted Gly178 and Met228 via the benzimidazole nitrogens. In the crystal structure, **22** rotated away from Met228 to interact with Leu177 and Gly178 via one benzimidazole nitrogen and the methylamine tail. The second benzimidazole nitrogen hydrogen-bonded with two ordered waters; these water molecules were not modeled in the docking. Finally, the docking prediction for **20** **(** **Figure 8** **D**) was inconsistent with the crystal structure (r.m.s.d. 3.1 Å). In the docking pose, the nitrogen on the imidazo ring interacted with Asp233 while the amine made contact with Gly178. This fragment had two configurations in the electron density, both modeled at 50% occupancy and neither of them resembling the docking pose.

## Discussion

Both bulk and ordered solvent effects play crucial roles in ligand binding [34]–[37], and this has motivated the development of methods to model ordered water molecules in molecular design [38]–[46]. Disentangling bulk contributions from those of ordered water molecules, and from the other convoluted terms encountered in biologically relevant target sites, has remained challenging. Because of its simplicity, the ability to determine structures to high resolution, the ability to seek and test new molecules prospectively, and to compare results with an analogous site that is closed to the bulk, the Gateless cavity seems well-suited to testing specific solvent-derived terms in protein ligand binding. Three key observations emerge from this study. First, and contrary to our own expectations, opening the cavity to bulk solvent has no general effect on the relative affinities of cationic and neutral ligands; the former continue to bind much more strongly, with the latter barely measurable. The effects on ligand affinity and binding geometry were context-dependent, and whereas several cationic ligands bound weaker to the opened Gateless cavity than to the analogous closed cavity (W191G), one cationic ligand had better affinity, as did one neutral ligand. The one common theme only emerges from the structures of the ligand-cavity complexes: those ligands that maintained their interactions with the anchoring aspartate and, at the same time, increased interactions with the new water channel, saw their affinity increase or stay the same. Ligands that could not do both changed their binding modes to favor a larger interface with the new water network, and saw their affinities drop (presumably they would have dropped further still had they maintained their closed-cavity geometries). Second, and again to our surprise, docking predictions broadly tracked these empirical trends, and as we moved to a more sophisticated physical model of ligand desolvation did so better still. In a prospective screen using this more sophisticated solvation term, the docking hit rate was high, with 14 of 15 ligands tested confirmed experimentally. The predicted and experimentally determined ligand geometries corresponded well for 4 of the 6 structures. Third, structures where we observed a substantial discrepancy between the predicted and experimental ligand poses saw the intervention of new ordered water molecules that we had not anticipated in the docking model.

The loss of affinity for compounds **1**, **2**, and **4**, with their flipped geometries in the site, versus the improved affinities for compounds **3** and **6**, and their conserved geometries, points to two effects that in these matched cavities perhaps may be disentangled. The opening to bulk solvent and the appearance of the new water network may reflect an improved solvation of the cavity in the Gateless mutant relative to that of W191G, reducing the relative free energy of the apo Gateless site. Conversely, in the closed loop W191G, the waters that provide the “chimney” from the site to the bulk, with which the ligands interact in the Gateless cavity, cannot bind as they are sterically excluded by the loop itself. This effect, in itself, likely reduces the affinity of all the ligands, which must now compete with lower energy solvent. This may also explain why the weakened ligands, like compound **1**, flip away from the anchoring aspartate: this allows interactions with the new water network precluded by their interactions with the aspartate, which effectively buried their polar groups, and better solvates the aspartate itself. Ligands like **3** and **6**, on the other hand, can adopt geometries that allow for extensive interface with the new water network, gaining interactions they did not make previously (or diminishing their own desolvation), while maintaining their original salt-bridge with the aspartate. Viewed another way, both ligands and the cavity may be optimizing their interaction with bulk solvent, for which the ordered waters are simply a visible proxy. Because all six ligands adopt geometries that seem to optimize interactions with the water network, we ourselves favor the more atomistic explanation. In either case, these observations illustrate how interactions with water can both compete with ligand-protein interactions – weakening net affinities and changing binding geometries – or complement them, improving affinity, depending on the particular features of the ligands. It is also illuminating that despite orientations of cationic ligands that apparently disrupt a crucial salt-bridge, the electrostatic interaction between the aspartate and the ligands is still maintained, and likely still the single most important contributor to recognition. This is reflected in the much higher affinity of cationic ligands over neutral ones, the failure of any newly docked neutral ligands to measurably bind to the cavity, and the high affinities of newly prioritized cationic ligands. Methods that base interaction energies on hydrogen-bond inventories, rather than overall electrostatics, may miss these contributions (DOCK 3.6 [32], [47], [48], like related physics-based approaches [49]–[55], uses a probe-charge model against an electrostatic potential map, and so does not depend on direct ligand-protein contacts, but rather electrostatic complementarity to an overall receptor potential).

In addition to asking how opening a cavity to solvent affects ligand recognition, a key part of this study was testing how docking would track the changes in site environment. In particular, we were interested in evaluating a new “Solvent-Excluded Volume” (SEV) method to treat ligand desolvation on docking [32]. This method should in principle better calculate partial ligand desolvation (i.e., retention of bulk solvent interaction) upon binding than our previous model, though neither considers ordered waters. In a large library screen, the older, less physical desolvation model highly-ranked mono-cations as likely molecules for the closed cavity, as is appropriate. However, against the open cavity about 20% of the top-ranked molecules were neutral; this reflects the high de-solvation penalty for cations, which are modeled as being almost entirely desolvated even in the open cavity. Conversely, with the new SEV method mono-cations entirely dominated the hit list (**Figure S3**); this reflects their substantially lower desolvation costs in the new method, owing to their retention of a substantial bulk solvation energy (see above), and consequent relative advantages over neutral molecules. We prospectively tested its performance in three ways: by testing three neutral molecules that the older method had prioritized (ranked 395, 493 and 500 out of 534,000 screened) but that the SEV method had deprioritized (ranked 2389, 2612 and 2950) (**Figure 5**), by testing 15 new molecules highly-ranked by the SEV method against the open cavity (**Figure 6**), and by determining X-ray crystal structures of six new ligands.

Consistent with the new solvation treatment, and inconsistent with the older method, the three neutral molecules were not observed to bind to the cavity at concentrations up to 1 mM. Conversely, 14 of the 15 new molecules highly ranked in the docking screen by the SEV solvation model, all cations, were found to bind with K_D_ values ranging from 8 to 820 µM and with most binding at better than 50 µM. Whereas these affinities may seem modest, the ligands are small–indeed almost always smaller than the neutral molecules tested–with ligand efficiencies as high as 0.66 kcal/mol/atom; the K_D_ values are competitive with the best observed in protein cavities of this size. Among the six ligands that were crystallized in complex with the protein, four adopted essentially the same pose in docking and crystal structure, with r.m.s.d. values of less than 1.2 Å and all interactions conserved (**10**, **14**, **17** and **24**, **Figure 8**). A fifth compound, **22**, had a higher r.m.s.d. of 1.4 Å, and made different direct contacts in the X-ray and docking structures. Intriguingly, this ligand was neither predicted by docking nor observed by crystallography to interact directly with Asp233 (**Figure 8** **E**). Finally, for one compound docking and crystallographic poses clearly disagreed (**20**, **Figure 8** **D**). In both of these last two cases, the presence of ordered water molecules in the binding site may explain reduced fidelity of the docking. Compound **22** interacts with two ordered waters that were not modeled in the docking and the ligand is rotated as compared to the dock pose. Compound **20** does not interact directly with water molecules, but the charged moiety on the ligand interacts with Gly178, which is itself involved in the new ordered water network. Here again, the role of the ordered waters, which we did not explicitly model, is highlighted.

The theoretical basis of ordered and bulk water effects on binding have been previously explored [34], [35], [39], [56]–[62], and there is a substantial body of empirical observations in this area [63]–[67]; what is new here is the engineering of two simple cavities, one a perturbation of the other, where these effects can be at least partially isolated. The closed (W191G) and opened Gateless (P190G/W191G/Δ192-3) cavities in Cytochrome *c* Peroxidase conserve most features that dominate ligand recognition – the opening of the cavity only deletes residues that are distal to the recognition features of the site, and the crucial cation-recognizing Asp235 is conserved in both (Asp233 in Gateless). Still, the effects of the substitution on ligands that bind to both targets are substantial, both in binding energy and in the structure of the ligand complexes. Part of the effects of opening the cavity to bulk solvent appears to be qualitatively captured by a continuum-based electrostatic model [68]–[71] in docking, though the role of ordered waters is not, and their effects can be considerable. In these model binding sites, one can hope to tease these contributions apart, and test any theory to treat them prospectively. These cavities are freely available to the community, and we hope that they may find use in exploring these and related questions in docking and molecular recognition.

## Materials and Methods

### Protein Preparation

The plasmid for the CcP-GA mutant protein was expressed and purified to apparent homogeneity as described [30].

### Molecules Tested

Compounds **1**, **2**, **3**, **5** and **6** were purchased from Aldrich, compounds **4**, **10**, **11**, **12**, **14**, **21** and **23** were purchased from Specs, compounds **7** and **9** were purchased from Molport, compound **8** was purchased from Vitas-M, compounds **15**, **18**, **20** and **22** were purchased from Enamine, compounds **17** and **24** were purchased from Adesis and compounds **13**, **16** and **19** were obtained from NCI. All molecules were used as supplied; suppliers confirm ≥95% purity for all compounds and compound identities for 12 of these were confirmed as relevant by subsequent x-ray crystallography.

### Crystallography

Compounds **1**, **2**, **3**, **4**, **5** and **6** were soaked into crystals at concentrations up to 50–100 mM in 25% MPD, or 25% MPD 10 mM MES for **10** [30]. Compounds **14**, **17**, **20**, **22**, and **24** were soaked at 100 mM in 25% MPD, into crystals grown under previously published conditions [72]. The electron density for the ligands was unambiguous in both the initial Fo-Fc and in the final 2Fo-Fc maps (**Figures 3** and **7**). In most structures, the shortened capping loop (Gly189-N194) was highly flexible and only the main conformation was modeled.

### Binding Measured by Titration of the UV-Vis Heme Soret Band

The compounds were tested for binding by measuring perturbation of the Heme Soret band at 10°C in 100 mM citrate buffer at pH 4.5 or 500 mM MES buffer pH 6.0 [27], [73]. To avoid competition with small cations like potassium, the pH of both buffer conditions was adjusted with Bis-Tris-Propane [27]. Stock solutions were made up in DMSO and diluted into assay buffer to derive K_D_ values in titration curves. K_D_ values were obtained by fitting the difference absorbance of the Heme Soret band to a one-site binding hyperbola in GraphPad Prism (GraphPad Software, Inc.).

### Low C-value Isothermal Titration Calorimetry

Experiments were performed as described [28]. Assays were performed at 10°C in 100 mM citrate buffer at pH 4.5. Ligand stocks were prepared in buffer from overnight dialysis of the protein to prevent buffer mismatch.

### Preparation of Fragment Set for Docking

The fragment sets were prepared using the standard ligand preparation protocol used for ligands in the ZINC database [31]. Molecules were protonated assuming a pH of 6.0 to minimize falsely cationic molecules owing to inaccuracies in the pKa calculations (**Text S1**).

### Docking

Docking calculations were carried out with DOCK3.6 [32], [47], [48] and DOCK3.54 [20], [48], [74]using a 1.74 Å crystallographic structure of Cytochrome *c* Peroxidase (PDB code 1KXM [30]) (**Text S2**).

## Supporting Information

Figure S1 Typical plot of a UV-Vis Heme Soret band titration (compound 10, KD 64 µM).
(TIF)

Figure S2 Log AUC curve for known CcP Gateless binders.
(TIF)

Figure S3 Charge distribution for the top 5000 docked molecules with old and new solvation maps. Dark grey: Previous full solvation map; Light grey: New Solvent-Exluded Volume (SEV) solvation map.
(TIF)

Table S1 ITC binding data for compounds **1**, **2**, **3**, **4** and **6** against CcP Gateless.
(DOCX)

Table S2 X-Ray data collection and refinement statistics.
(DOCX)

Text S1 Preparation of fragment set for docking.
(DOCX)

Text S2 Docking.
(DOCX)
