# Supplementary figures and images for: Roles for Ordered and Bulk Solvent in Ligand Recognition and Docking in Two Related Cavities
Source: PLoS One. 2013 Jul 18;8(7):e69153. doi: 10.1371/journal.pone.0069153 (PMC3715451; doi:10.1371/journal.pone.0069153)

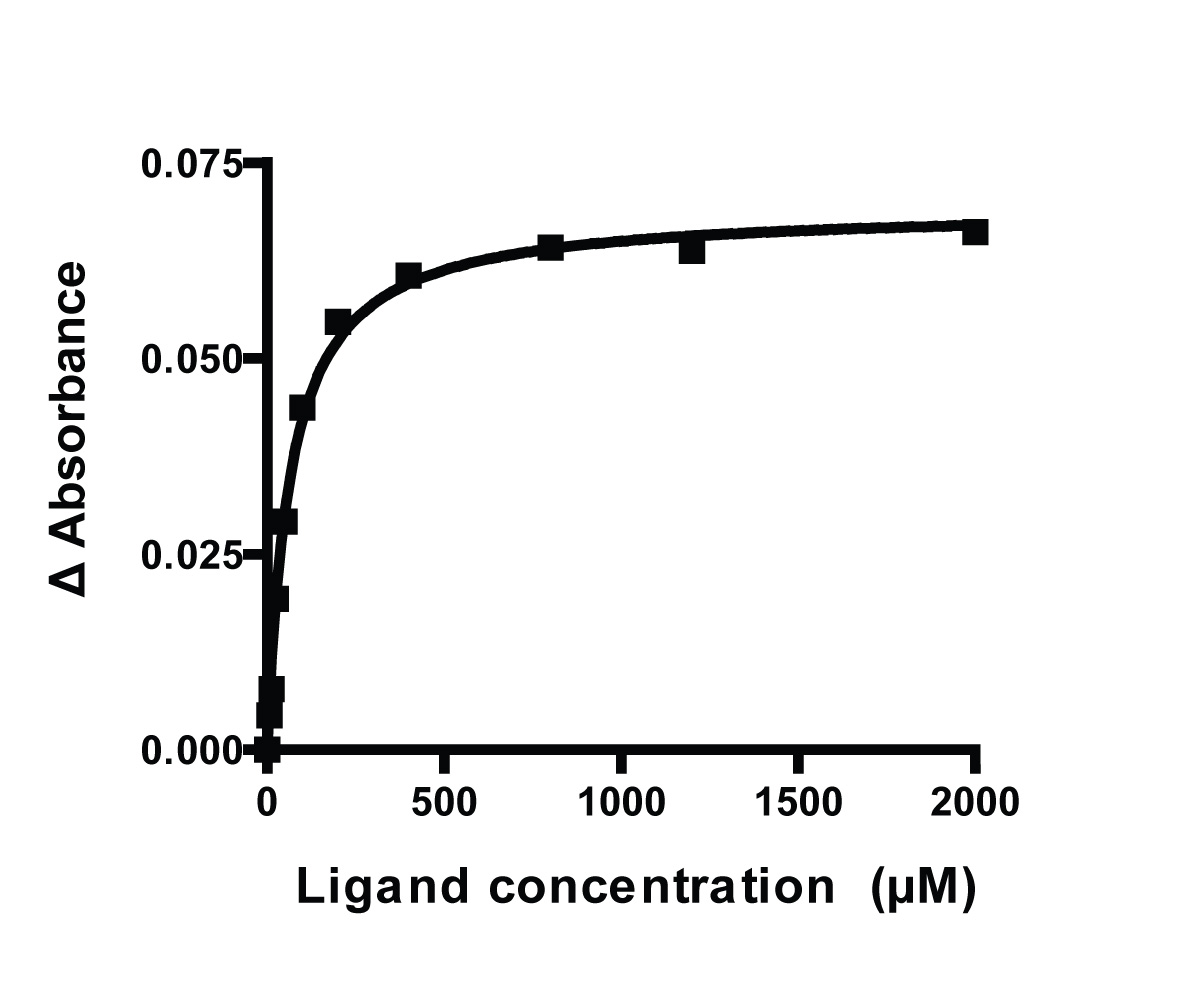

Supplement: Figure S1 — Typical plot of a UV-Vis Heme Soret band titration (compound 10, KD 64 µM). (TIF) [file pone.0069153.s001.tif]

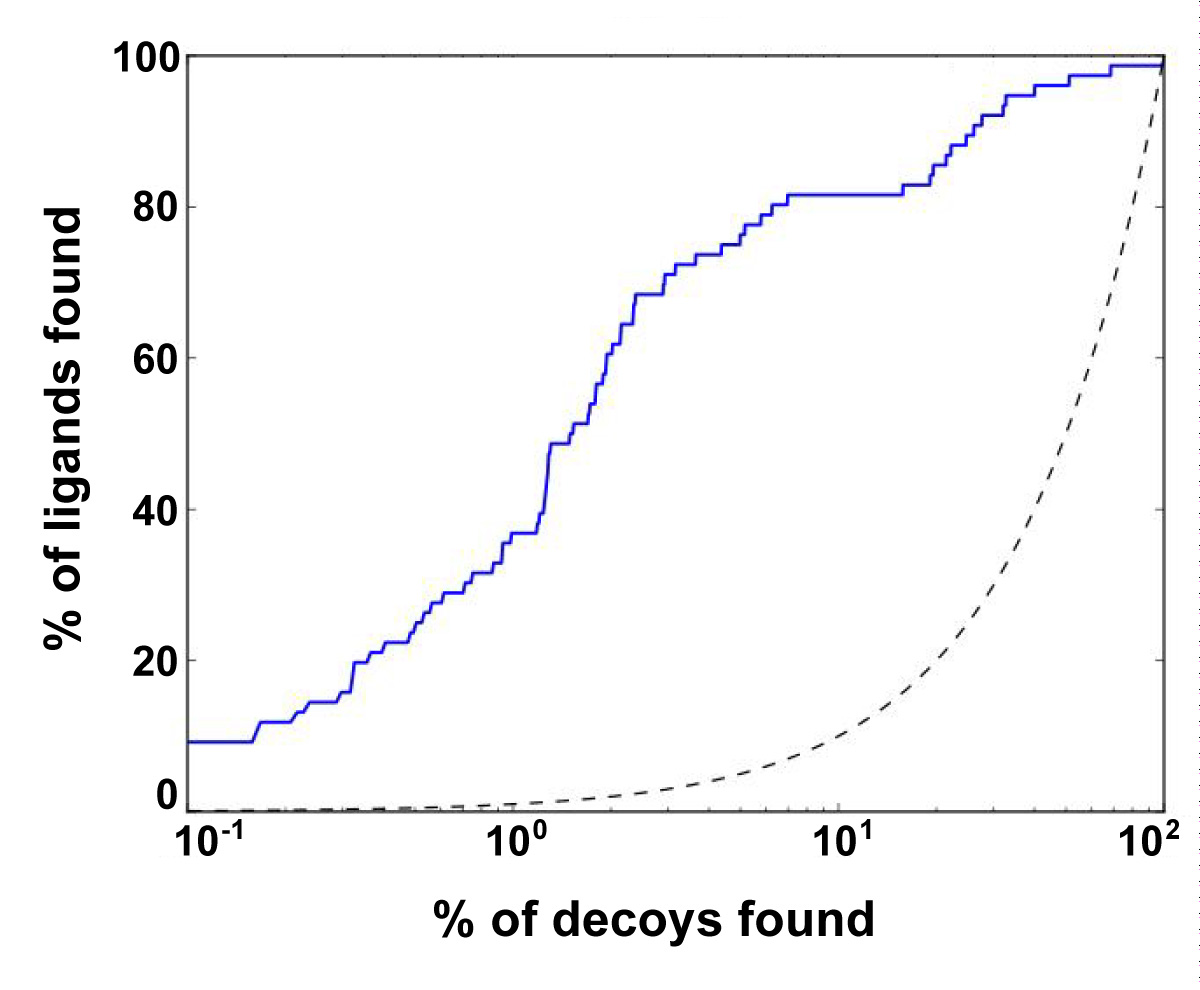

Supplement: Figure S2 — Log AUC curve for known CcP Gateless binders. (TIF) [file pone.0069153.s002.tif]

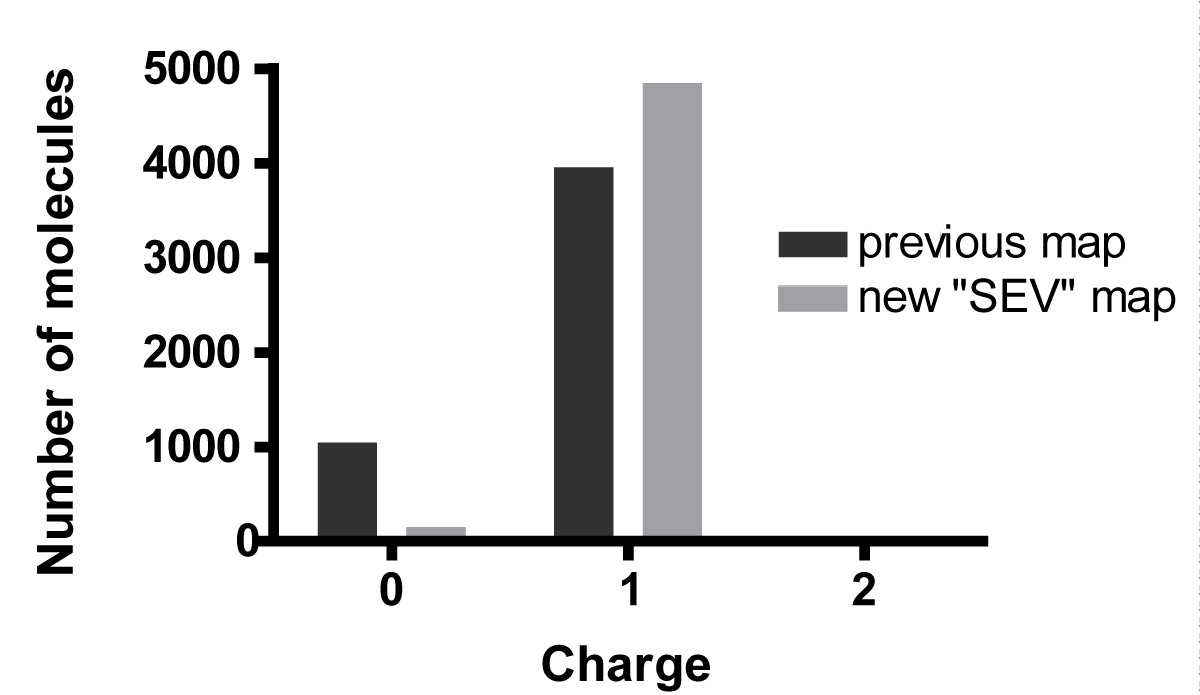

Supplement: Figure S3 — Charge distribution for the top 5000 docked molecules with old and new solvation maps. Dark grey: Previous full solvation map; Light grey: New Solvent-Exluded Volume (SEV) solvation map. (TIF) [file pone.0069153.s003.tif]
