# Supplementary material for: Roles for Ordered and Bulk Solvent in Ligand Recognition and Docking in Two Related Cavities
Source: PLoS One. 2013 Jul 18;8(7):e69153. doi: 10.1371/journal.pone.0069153 (PMC3715451; doi:10.1371/journal.pone.0069153)
Supplement: Table S1 — ITC binding data for compounds 1, 2, 3, 4 and 6 against CcP Gateless. (DOCX) [file pone.0069153.s004.docx]

### Table S1. ITC data (ΔG, ΔH and ΔS): binding of compounds 1, 2, 3, 4 and 6 to CcP Gateless at 283K.

| **ID** | **Chemical name** | **ΔG** (kcal/mol) | **ΔH** (kcal/mol) | **TΔS** (kcal/mol) |
| --- | --- | --- | --- | --- |
| **1** | 2-amino-5-methylthiazole | -5.21 | -5.00 | 0.21 |
| **2** | 2,4-diaminopyrimidine | -4.42 | -5.56 | -1.14 |
| **3** | 2,6-diaminopyridine | -5.73 | -9.04 | -3.31 |
| **4** | 3-amino-1-methylpyridinium | -3.6† | -7.2† | -3.6† |
| **6** | 3-fluorocatechol | -3.2† | -1.0† | -7.2† |

† Approximate value determined by partial ITC curves—assessment of these compounds was limited by solubility.
