# Supplementary material for: Roles for Ordered and Bulk Solvent in Ligand Recognition and Docking in Two Related Cavities
Source: PLoS One. 2013 Jul 18;8(7):e69153. doi: 10.1371/journal.pone.0069153 (PMC3715451; doi:10.1371/journal.pone.0069153)
Supplement: Table S2 — X-Ray data collection and refinement statistics. (DOCX) [file pone.0069153.s005.docx]

### Table S2. X-Ray data collection and refinement statistics.

| **PDB ID** | 4JM5 | 4JM6 | 4JM8 | 4JM9 |
| --- | --- | --- | --- | --- |
| **Compound #** | **1** | **2** | **3** | **4** |
| **Ligand name** | 2-amino-5-methylthiazole | 2,4-diaminopyrimidine | 2,6-diaminopyridine | 3-amino-1-methylpyridinium |
| **ZINC ID** | C08652421 | C01661391 | C00333587 | C00335002 |
| **Data collection** |  |  |  |  |
| Space group | P212121 | P212121 | P212121 | P212121 |
| Cell dimensions |  |  |  |  |
| *a*, *b*, *c* (Å) | 51.31 74.94 106.64 | 50.85 75.58 106.69 | 50.84 74.52 106.46 | 51.03 74.70 106.59 |
| α, β, γ (°) | 90, 90, 90 | 90, 90, 90 | 90, 90, 90 | 90, 90, 90 |
| Resolution (Å) | 61.31-1.26  (1.29-1.26) | 61.66-1.45  (1.50-1.45) | 61.08-1.30  (1.35-1.30) | 61.20-1.41  (1.46-1.41) |
| *R*_merge_ | 0.064 (0.423) | 0.073 (0.343) | 0.073 (0.248) | 0.105 (0.345) |
| *I* / σ*I* | 15.0 (2.5) | 20.1 (2.8) | 16.9 (5.0) | 15.3 (6.5) |
| Completeness (%) | 97.1 (78.8) | 98.5 (86.2) | 98.3 (87.8) | 99.3 (94.0) |
| Redundancy | 4.7 (3.8) | 4.7 (3.8) | 4.6 (3.7) | 4.9 (4.7) |
|  |  |  |  |  |
| **Refinement** |  |  |  |  |
| Resolution (Å) | 61.31-1.26  (1.29-1.26) | 61.66-1.45  (1.49-1.45) | 61.08-1.30  (1.33-1.30) | 61.20-1.41  (1.44-1.41) |
| No. reflections | 102683 (5993) | 68527 (4376) | 93366 (6081) | 75167 (5192) |
| *R*_work_ / *R*_free_ | 0.1276/ 0.1547 | 0.1148/ 0.1448 | 0.1170/ 0.1368 | 0.1149/ 0.1391 |
| No. atoms | 3109 | 3152 | 3366 | 3402 |
| Average B-factor | 19.6 | 14.6 | 15.4 | 14.9 |
| R.m.s. deviations |  |  |  |  |
| Bond lengths (Å) | 0.019 | 0.019 | 0.019 | 0.018 |
| Bond angles (°) | 1.83 | 1.74 | 1.69 | 1.67 |

*Values in parentheses are for highest-resolution shell.

| **PDB ID** | 4JMW | 4JMA | 4JMB | 4JMS |
| --- | --- | --- | --- | --- |
| **Compound #** | **5** | **6** | **10** | **14** |
| **Ligand name** | phenol | 3-fluorocatechol | 5,6,7,8-tetrahydrothieno[2,3-b]quinolin-4-amine | imidazo[1,2-a]pyridin-5-amine |
| **ZINC ID** | C05133329 | C00164683 | C00346401 | C00337657 |
| **Data collection** |  |  |  |  |
| Space group | P212121 | P212121 | P212121 | P212121 |
| Cell dimensions |  |  |  |  |
| *a*, *b*, *c* (Å) | 51.05 74.41 106.60 | 51.07 74.65 106.42 | 50.97 74.80 106.82 | 51.17 75.07 106.12 |
| α, β, γ (°) | 90, 90, 90 | 90, 90, 90 | 90, 90, 90 | 90, 90, 90 |
| Resolution (Å) | 39.15-1.19  (1.22-1.19) | 61.08-1.60  (1.64-1.60) | 30-1.30  (1.33-1.30) | 30-1.75  (1.80-1.75) |
| *R*_merge_ | 0.041 (0.432) | 0.046 (0.101) | 0.052 (0.436) | 0.083 (0.495) |
| *I* / σ*I* | 18.4 (2.2) | 43.8 (22.3) | 13.8 (3.1) | 14.0 (2.6) |
| Completeness (%) | 95.2 (66.3) | 97.5 (99.2) | 99.5 (99.9) | 99.1 (97.6) |
| Redundancy | 4.1 (2.4) | 3.4 (3.4) | 4.2 (4.1) | 4.4 (4.5) |
|  |  |  |  |  |
| **Refinement** |  |  |  |  |
| Resolution (Å) | 39.15-1.19  (1.22-1.19) | 61.08-1.60  (1.64-1.60) | 29.19-1.30  (1.33-1.30) | 29.1-1.75  (1.80-1.75) |
| No. reflections | 117724 (5965) | 47798 (3475) | 100525 (7406) | 41638 (3002) |
| *R*_work_ / *R*_free_ | 0.1254/ 0.1514 | 0.1408/ 0.1618 | 0.1366/ 0.1540 | 0.1346/ 0.1660 |
| No. atoms | 3304 | 3043 | 2953 | 3010 |
| Average B-factor | 15.0 | 13.2 | 11.8 | 15.1 |
| R.m.s. deviations |  |  |  |  |
| Bond lengths (Å) | 0.019 | 0.018 | 0.005 | 0.017 |
| Bond angles (°) | 1.80 | 1.63 | 0.99 | 2.6 |

*Values in parentheses are for highest-resolution shell.

| **PDB ID** | 4JMT | 4JMV | 4JMZ | 4JN0 |
| --- | --- | --- | --- | --- |
| **Compound #** | **17** | **20** | **22** | **24** |
| **Ligand name** | (1H-Pyrrolo[3,2-b] pyridin-6-yl)methanol | Imidazo[1,2-a]pyridin-6-amine | N-methyl-1H-1,3-benzodiazol-2-amine | 1H-Pyrrolo[3,2-b] pyridine-6-carbaldehyde |
| **ZINC ID** | C14401114 | C18555973 | C00039693 | C14401108 |
| **Data collection** |  |  |  |  |
| Space group | P212121 | P212121 | P212121 | P212121 |
| Cell dimensions |  |  |  |  |
| *a*, *b*, *c* (Å) | 51.30 74.58 106.47 | 51.23 73.84 106.56 | 51.18 74.57 105.15 | 50.90 74.56 106.07 |
| α, β, γ (°) | 90, 90, 90 | 90, 90, 90 | 90, 90, 90 | 90, 90, 90 |
| Resolution (Å) | 30-1.60  (1.64-1.60) | 30-1.82  (1.87-1.82) | 30-1.82  (1.87-1.82) | 30-1.86  (1.91-1.86) |
| *R*_merge_ | 0.063 (0.464) | 0.059 (0.464) | 0.087 (0.496) | 0.08 (0.483) |
| *I* / σ*I* | 15.5 (2.8) | 18.7 (3.0) | 12.6 (2.9) | 15.7 (2.9) |
| Completeness (%) | 98.8 (97.2) | 99.5 (97.4) | 99.8 (99.9) | 99.1 (98.2) |
| Redundancy | 4.3 (4.2) | 4.5 (4.2) | 4.4 (4.5) | 4.1 (4.1) |
|  |  |  |  |  |
| **Refinement** |  |  |  |  |
| Resolution (Å) | 29.2-1.60  (1.64-1.60) | 29.2-1.82  (1.87-1.82) | 29-1.82  (1.87-1.82) | 29-1.86  (1.91-1.86) |
| No. reflections | 54010 (3885) | 36829 (2595) | 36761 (2671) | 34149 (2471) |
| *R*_work_ / *R*_free_ | 0.1576/ 0.1752 | 0.1394/ 0.1745 | 0.1449/ 0.1816 | 0.1498/ 0.1942 |
| No. atoms | 2907 | 2896 | 2956 | 2849 |
| Average B-factor | 13.3 | 19.74 | 17.80 | 14.5 |
| R.m.s. deviations |  |  |  |  |
| Bond lengths (Å) | 0.006 | 0.014 | 0.014 | 0.006 |
| Bond angles (°) | 0.99 | 2.37 | 2.40 | 0.93 |

*Values in parentheses are for highest-resolution shell.
