# Supplementary material for: Roles for Ordered and Bulk Solvent in Ligand Recognition and Docking in Two Related Cavities
Source: PLoS One. 2013 Jul 18;8(7):e69153. doi: 10.1371/journal.pone.0069153 (PMC3715451; doi:10.1371/journal.pone.0069153)
Supplement: Text S1 — Preparation of fragment set for docking. (DOCX) [file pone.0069153.s006.docx]

### Supplementary Text S1. Preparation of fragment set for docking.

The fragment subset contained about 534,000 molecules and was prepared from the ZINC library[[1](#_ENREF_1),[2](#_ENREF_2)] by filtering for compounds with molecular weight between 30 and 250 Da. Each molecule was prepared for docking using the program OMEGA[[3](#_ENREF_3)]. Partial atomic charges and transfer free energies were calculated using AMSOL[[4](#_ENREF_4),[5](#_ENREF_5)] and van der Waals parameters were derived from an all-atom AMBER potential[[6](#_ENREF_6)]. For the first round of docking the target pH of the database was set to 4.5 (pH of the assay conditions) with a tolerance of +/- 1 pH unit (Epik software package, Schrödinger Inc). For the second round of docking, after it became apparent that many molecules included in the initial docking were not present in their charged form at sufficient concentration in the assay conditions, the target pH was set to 6 +/- 0.75 to ensure the ligands were correctly charged, both in the assay and in the crystallographic conditions. Fifteen compounds were selected among the top 500 molecules, purchased and tested experimentally. To evaluate the accuracy of the docking, the predicted poses were overlaid onto the crystallographic conformations, using protein backbone and ligand atoms. R.m.s.d. values were calculated by using ligand atoms only.

1. Irwin JJ, Shoichet BK (2005) ZINC - A free database of commercially available compounds for virtual screening. Journal of Chemical Information and Modeling 45: 177-182.

2. Irwin JJ, Sterling T, Mysinger MM, Bolstad ES, Coleman RG (2012) ZINC: A Free Tool to Discover Chemistry for Biology. Journal of Chemical Information and Modeling 52: 1757-1768.

3. Boström J, Greenwood JR, Gottfries J (2003) Assessing the performance of OMEGA with respect to retrieving bioactive conformations. Journal of Molecular Graphics and Modelling 21: 449-462.

4. Chambers CC, Hawkins GD, Cramer CJ, Truhlar DG (1996) Model for Aqueous Solvation Based on Class IV Atomic Charges and First Solvation Shell Effects. The Journal of Physical Chemistry 100: 16385-16398.

5. Li J, Zhu T, Cramer CJ, Truhlar DG (1998) New Class IV Charge Model for Extracting Accurate Partial Charges from Wave Functions. The Journal of Physical Chemistry A 102: 1820-1831.

6. Weiner SJ, Kollman PA, Nguyen DT, Case DA (1986) An all atom force field for simulations of proteins and nucleic acids. Journal of Computational Chemistry 7: 230-252.
