# Supplementary material for: Roles for Ordered and Bulk Solvent in Ligand Recognition and Docking in Two Related Cavities
Source: PLoS One. 2013 Jul 18;8(7):e69153. doi: 10.1371/journal.pone.0069153 (PMC3715451; doi:10.1371/journal.pone.0069153)
Supplement: Text S2 — Docking. (DOCX) [file pone.0069153.s007.docx]

### Supplementary Text S2. Docking.

The receptor structure was prepared by removing all non-protein atoms and the protonation states of ionizable residues were set to the most probable in aqueous solution at pH 7 and protonated using Sybyl. Sixty matching spheres were used, and these were either based on the atoms of the crystallographic ligands or positioned manually. The spheres were also labeled for chemical matching based on the local receptor environment[[1](#_ENREF_1)]. Bin size, bin size overlap, and distance tolerance were set to 0.2, 0.2, and 0.75 Å, respectively, for both the binding site matching spheres and the docked molecules. The electrostatic potential in the binding site was prepared using the program Delphi[[2](#_ENREF_2)] and the van der Waals grid by CHEMGRID[[3](#_ENREF_3)]. The electrostatic interaction energies were corrected for ligand desolvation. This was done either using a full desolvation model in DOCK3.54 or a solvation model derived from the low dielectric volume occupied by the protein in the region of the binding site, in DOCK3.6 [[4](#_ENREF_4),[5](#_ENREF_5)]. Partial charges from the united-atom AMBER force field[[6](#_ENREF_6)] were used for all receptor atoms except for Asp233, for which the dipole moment was increased to favor hydrogen bonding with docked fragments, as previously published. On average, 239 orientations were calculated for each of the 534,000 ZINC molecules screened, and an average of 31578 conformations of each molecule was scored (a total of 3918 complexes were evaluated per molecule).

1. Shoichet BK, Kuntz ID (1993) Matching chemistry and shape in molecular docking. Protein Eng 6: 723-732.

2. Nicholls A, Honig B (1991) A rapid finite difference algorithm, utilizing successive over-relaxation to solve the Poisson–Boltzmann equation. Journal of Computational Chemistry 12: 435-445.

3. Meng EC, Shoichet BK, Kuntz ID (1992) Automated docking with grid-based energy evaluation. Journal of Computational Chemistry 13: 505-524.

4. Wei BQQ, Baase WA, Weaver LH, Matthews BW, Shoichet BK (2002) A model binding site for testing scoring functions in molecular docking. Journal of Molecular Biology 322: 339-355.

5. Mysinger MM, Shoichet BK (2010) Rapid context-dependent ligand desolvation in molecular docking. J Chem Inf Model 50: 1561-1573.

6. Case DA, Darden TA, Cheatham TE, Simmerling CL, Wang J, et al. (2012) AMBER 12. University of California, San Francisco
